# The role of neutrophil extracellular traps in acute lung injury

**DOI:** 10.3389/fimmu.2022.953195

**Published:** 2022-07-29

**Authors:** Davide Scozzi, Fuyi Liao, Alexander S. Krupnick, Daniel Kreisel, Andrew E. Gelman

**Affiliations:** ^1^ Department of Surgery, Washington University in St. Louis, St. Louis, MO, United States; ^2^ Department of Surgery, University of Maryland, Baltimore, MD, United States; ^3^ Department of Pathology and Immunology, Washington University in St. Louis, St. Louis, MO, United States

**Keywords:** NETs (neutrophil extracellular traps), ALI (acute lung injury), ARDS (acute respiratory distress syndrome), sterile inflammatory response, infections and sepsis, COVID-19, DAMPs (damage-associated molecular patterns), Thromboinflammation

## Abstract

Acute lung injury (ALI) is a heterogeneous inflammatory condition associated with high morbidity and mortality. Neutrophils play a key role in the development of different forms of ALI, and the release of neutrophil extracellular traps (NETs) is emerging as a common pathogenic mechanism. NETs are essential in controlling pathogens, and their defective release or increased degradation leads to a higher risk of infection. However, NETs also contain several pro-inflammatory and cytotoxic molecules than can exacerbate thromboinflammation and lung tissue injury. To reduce NET-mediated lung damage and inflammation, DNase is frequently used in preclinical models of ALI due to its capability of digesting NET DNA scaffold. Moreover, recent advances in neutrophil biology led to the development of selective NET inhibitors, which also appear to reduce ALI in experimental models. Here we provide an overview of the role of NETs in different forms of ALI discussing existing gaps in our knowledge and novel therapeutic approaches to modulate their impact on lung injury.

## Introduction

ALI is an inflammatory condition characterized by the acute onset of lung tissue damage and pulmonary dysfunction originating from infectious or sterile insults (1). Typical features of ALI are the alveolar accumulation of protein-rich fluid and activated immune cells due to pulmonary endothelial barrier disruption and increased vascular permeability (2). The pathological alterations of ALI are responsible for a clinical syndrome characterized by extensive non-cardiogenic pulmonary edema and decreasing oxygenation, also known as acute respiratory distress syndrome (ARDS) (3). ARDS is associated with high morbidity and mortality (~40%), and its increasing incidence, particularly related to the recent COVID-19 pandemic, represents a significant global burden (4).

Airway neutrophilia has been historically considered a hallmark of ARDS (5). However, the underlying mechanisms that control neutrophil contribution to ALI are not fully understood. Although neutrophils play well-established functions in regulating pulmonary injury through the generation of reactive oxygen species (ROS) or conducting phagocytosis and degranulation, recent reports have highlighted a critical role for NETosis in ALI pathogenesis. NETs consist of a mix of nuclear chromatin (6), mitochondrial DNA (7–9) and neutrophil granule proteins (10) that primarily absolve a defensive role against lung infections. On the other hand, accumulating evidence indicate that NETosis is also increased in lung sterile inflammatory conditions and that exuberant NET release promotes microvascular dysfunction, thromboinflammation, and direct cellular injury (11). Moreover, high levels of NETs in the peripheral blood or bronchoalveolar lavage (BAL) of critically ill subjects are frequently associated with the worst ARDS outcomes (12, 13).

**Figure 1:**
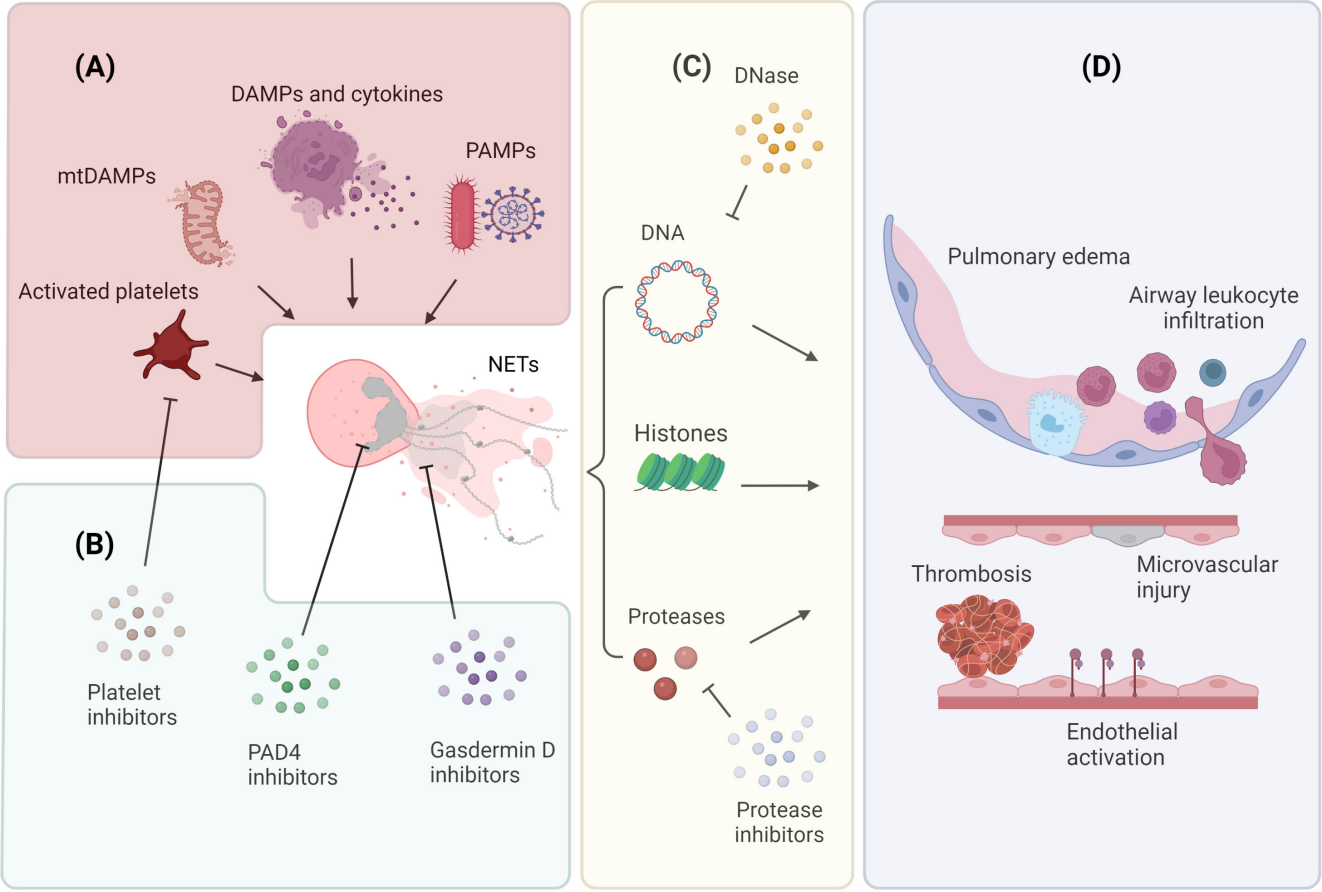
Mechanisms of NET release and NET-mediated lung injury **(A)** Neutrophils release NETs in response to endogenous and exogenous stimuli. Endogenous factors include DAMPs, pro-inflammatory cytokines, mtDAMPs, and molecules released by activated platelets; exogenous factors include PAMPs associated with microbial infections. **(B)** Inhibition of NET generation and release. The contribution of platelets to NETosis can be attenuated by using platelet activation inhibitors; neutrophil chromatin decondensation can be targeted by using PAD4 inhibitors; neutrophil membrane permeabilization can be prevented by using gasderimin D inhibitors. **(C)** NETs comprise a DNA scaffold decorated with granule proteases and histone proteins. NET DNA scaffold can be digested by DNase; NET proteolytic activity can be abrogated by specific protease inhibitors. **(D)** NETs release contributes to the pathogenesis of ALI. NETs facilitate the formation of thrombi, promote endothelial cell activation, and induce microvascular injury. These microvascular alterations result in increased vascular permeability, intra-alveolar accumulation of protein-rich fluid, and infiltration of inflammatory cells. Image created by DS using BioRender (https://biorender.com/).

**Figure 2:**
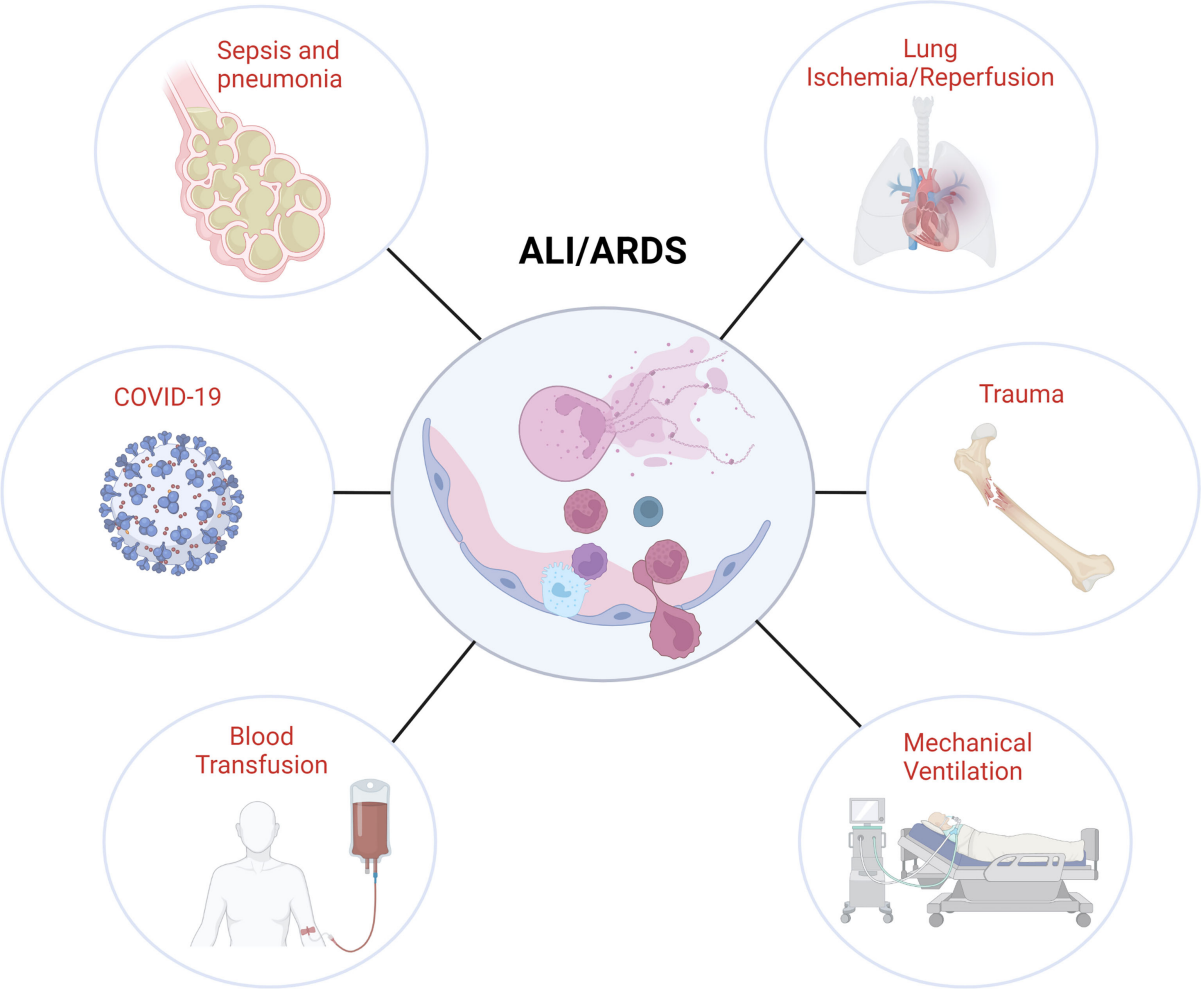
Association of NETs with different ALI/ARDS etiologies. Image created by DS using BioRender (https://biorender.com/).

In this review, we will discuss general mechanisms of NETosis and NET-mediated tissue damage (**Figure 1**) with a specific focus on the contribution of NETs to different forms of infective and sterile ALI (**Figure 2**).

## Non-vital and vital NETosis

NETosis was first described in 2004 when Brinkman and coworkers observed that activated neutrophils could kill bacteria by releasing nuclear chromatin decorated with proteins usually confined to their granules (14). Following studies characterized the NADPH oxidase 2 (NOX2) Ros-dependent pathway involved in the release of NETs and defined NETosis as a new mechanism of neutrophil programmed cell death distinct from apoptosis and necrosis (15, 16). NETosis was originally described as a slow multistep process that leads directly to senescence, where chromatin decondensation and nuclear envelope rupture allow for mixing nuclear material with cytoplasm content. Pore-forming proteins like gasdermin D then permeabilize the plasma membrane allowing for the release of NETs into the extracellular space (17). However, this view of NETosis as a terminal process has been recently challenged by the demonstration of an alternative, non-lytic and NADPH-independent mechanism of NET release, which appears to be particularly rapid in response to bacterial infection and leaves behind a neutrophil that can still exclude vital dyes (18). This form of NETosis is initiated by a sudden rise in intracellular calcium that leads to the expulsion of nuclear chromatin and granule proteins, resulting in an anucleated cytoplast still capable of migration and phagocytosis (11, 18, 19). Finally, a third type of fast, vital and ROS-dependent NETosis was recently described in response to granulocyte-macrophage colony-stimulating factor or lipopolysaccharide (LPS) leading to the release of mitochondrial DNA (mtDNA) mixed with granule proteins (7).

Almost two decades after the first observations, it is now commonly accepted that NET release can occur in response to multiple stimuli *via* several and frequently interconnected activation pathways (17, 20–22) that are not necessarily associated with neutrophil death (7, 18, 19). Regardless of the stimuli and the activation mechanisms, NET composition has intrinsic pro-inflammatory characteristics due to the presence of granule-derived proteases, histones and cell-free DNA (23).

## NET composition

### Serine proteases

Under homeostatic conditions, neutrophil azurophilic granules contain different serine proteases, including neutrophil elastase (NE), cathepsin G (CatG), proteinase 3 (PR3), and neutrophil serine protease 4 (NSP4). Due to their antimicrobial and immunomodulatory function, these pre-stored catalytically active mediators play a major contribution in the physiological response to infection (24). However, their uncontrolled extracellular release may have unintended consequences by causing damage to the surrounding healthy tissue (25). Proteomic analysis and studies on the functional activity of human NETs indicate that NE is the most abundant non-histone protein and the predominant responsible for the NET “proteolytic signature” (10, 26). NE is a well-established mediator of alveolo-capillary permeability where it is thought to trigger microvascular injury through primarily catalyzing endothelial cell cadherin proteolysis (27–29). In a mouse model of LPS induced endotoxemia, treatment with the NE selective inhibitor Sivelestat attenuated pulmonary endothelial injury reducing endothelial glycocalyx damage and preserving thrombomodulin and syndecan-1 expression (30). Similar findings were observed in work by Okeke and colleagues, where Sivelestat treatment reduced NET-mediated injury on human umbilical vein endothelial cells (HUVECs) (31). Interestingly, Sivelestat was able to reverse the NET-mediated upregulation of Intercellular Adhesion Molecule-1 (ICAM-1) on HUVECs, suggesting that NETs may propagate the inflammatory response of endothelial cells through NE (31). Furthermore, reduced NET deposition after Sivelestat treatment also suggested that NE may be directly involved in the release of NETs (31). This observation would be consistent with previous findings describing the role of NE nuclear translocation in facilitating histone degradation and chromatin decondensation (32). However, in mice genetically deficient for NE, the stimulation with the well-known NETosis inducer, phorbol 12-myristate 13-acetate (PMA), does not abrogate the release of NETs (33). In addition, the effect of NE inhibition appears to vary among neutrophils from different healthy donors, suggesting that NE may not always be necessary for the execution of NETosis (34).

Other proteases such as PR3 and Cat G are known to enhance inflammatory reactions by modulating the balance between pro and anti-inflammatory proteins (35). However, despite their documented proteolytic activity in human NETs, the precise contribution to mediating ALI is currently less clear.

In summary, besides the explicit role of NE in NET-mediated lung microvascular damage, the overall contribution of neutrophil serine proteases to NET-dependent inflammation in ALI is still undefined.

### Histones

Histones are generally located within the cell nucleus, where they play an essential role in organizing and regulating DNA (36). During NETosis, the enzyme peptidyl arginine deiminase 4 (PAD4) alters histone intermolecular interactions by converting arginine residues into citrulline (37). Citrullination decreases histone protein stability and facilitates chromatin decondensation and chromosomal DNA expulsion (38, 39). PAD4-dependent histones citrullination has been frequently reported as a critical component of NETosis in response to numerous physiological stimuli (40), and treatment with PAD4 inhibitors such as Cl-amidine (41) and GSK484 (42) have been shown positive effects in attenuating ALI. Interestingly, recent works indicate that there are conditions where NETosis can occur even in the absence of PAD4 activity, for example, in response to *Candida albicans*, *Klebsiella pneumoniae* or cholesterol crystals (43–45). These evidence suggest that during NETosis, chromatin decondensation may be not exclusively dependent on histone citrullination and that alternative mechanisms, such as direct histone cleavage by NE nuclear translocation, may also be involved (32). How different NET stimuli activate alternative mechanisms of chromatin decondensation and what is the level of redundancy and interaction between such pathways remains unclear, indicating the need for a better characterization of the upstream signaling involved in NET formation and release.

Extracellular histones account for ~70% of all NET-associated proteins (10), and their presence highly contributes to NET-mediated thromboinflammation (36, 46). For example, histone engagement of TLR2 and TLR4 can induce platelet activation resulting in the release of thrombin (47). In addition, histones can activate αIIbβ3 integrin on the platelet surface, inducing subsequent fibrinogen-mediated platelet aggregation (48). Histones also appear to stimulate TLR9-dependent responses leading to mitochondrial ROS production and NLR Family Pyrin Domain Containing 3 (NLRP3) inflammasome activation (49). In a mouse model of ALI secondary to severe trauma, high circulating histones were associated with signs of edema, increased alveolar wall thickening, and occasional hemorrhage (50). However, inhibition with TLR4 and TLR2-neutralizing antibodies did not show protective effects on cultured endothelial cells suggesting that TLR activation may not be the major pathway for histone toxicity. In this regard, extracellular histones have been shown to cause endothelial cell damage through direct interactions with the plasma membrane phospholipids, which lead to increased transmembrane conductance (50). Although DNase treatment is generally considered effective in degrading NET DNA scaffold, questions remain on the fate of NET proteins, particularly histones, after DNase digestion. For example, Saffarzadeh and coworkers show that DNase-mediated NET degradation was insufficient to abolish NET cytotoxicity, suggesting that histone proteins, after DNA digestion, can still mediate most of the NET-related tissue damage (51). Similar findings are also present in work by Kolaczkowska and colleagues where, in a model of bloodstream infection with methicillin-resistant Staphylococcus aureus (MRSA), they demonstrate that significant amount of active histones and NE remained attached to the vasculature wall after DNase treatment (52).

In summary, extracellular histones play a potent and heterogeneous contribution in NET-mediated thromboinflammation and tissue injury. Reducing the release, neutralizing, or blocking histone signal transduction may represent in the future a novel pharmacological approach for ALI treatment.

### DNA

DNA forms the NET backbone, and for a long time, its singular function has been thought limited to maintaining NET structural integrity. However, recent findings suggest that DNA may also play a direct role in promoting thromboinflammation. Besides providing a scaffold for the recruitment of platelets, leukocytes, and coagulation factors (53), cell-free DNA derived from NETs has been shown to mediate thrombin generation through the Factor XII- or Factor XI-dependent coagulation pathways in patients with sepsis (54). Accordingly, the digestion or precipitation of DNA networks markedly diminishes NET pro-coagulant effects (55). In addition to nuclear DNA, NETs can also be enriched with DNA derived from mitochondria (mtDNA) (7–9). mtDNA are circular molecules of double-stranded DNA that encode for some of the genes that form the electron transport chain as well as drive mitochondrial-specific protein synthesis (56). However, given their ancient bacterial origin (57), NET-associated mtDNA release can act as a damaged associated molecular pattern (DAMP) by stimulating the CpG DNA sensor TLR9 (58, 59) as well as triggering NLRP3 inflammasome pathway activation on immune cells (60). In addition, the NET-mediated release of oxidized mtDNA has been shown to stimulate type I interferon (IFN) signaling through a signaling pathway dependent on the DNA sensor stimulator of interferon genes (STING), an intracellular pattern recognition receptor (9). Moreover, recent findings indicate that mtDNA can also directly activate neutrophils to release NETs through TLR9 engagement (61–63).

In summary, besides acting as a scaffold, NET DNA may directly stimulate innate immune responses that promote pulmonary inflammation.

## NETs and ALI etiologies

### Sepsis and pneumonia

Sepsis is a common and deadly inflammatory condition defined as a dysregulated host response to infection associated with multi-organ dysfunction (64). With the development of ARDS, lung involvement is frequently observed in critically ill subjects affected with sepsis (65). Moreover, infectious pneumonia is one of the leading causes of sepsis and the most frequent cause of ALI (66–68). Although pathogens themselves can directly damage the lung, a significant contribution to the injury comes from the exuberant activation of the host immune cells in response to pathogen-associated molecular patterns (PAMPs) and DAMPs, collectively defined as “alarmins” (69–71).

NETs have been firstly described as a potent defensive mechanism able to block and inactivate pathogens preventing their growth and dissemination (14, 72). The cationic NETs components provide an ideal scaffold for electrostatic interactions with the negatively charged surface of microorganisms exposing them to a high local concentration of cytotoxic molecules. Moreover, even in the absence of direct killing, experiments in primary human neutrophils showed that NETs can indirectly increase the complement ability to destroy pathogens such as *Pseudomonas aeruginosa* and *Staphylococcus aureus* (73). Considering the major role played by NETs in controlling infections, it is not surprising that several microbes have evolved specific adaptive strategies to counteract this defensive mechanism (74). For example, *Streptococcus pneumoniae*, the most common cause of community-acquired pneumonia (CAP), expresses endonucleases that degrade the NET DNA scaffold allowing for bacterial escape (75). In alternative, *Bordetella pertussis*, the causative agent of whooping cough, expresses adenylate cyclase toxin (ACT) which inhibits the release of NETs by increasing cAMP levels and reducing intracellular ROS production (76). In humans, the critical role of NETs in protecting from lung infections is evident in subjects affected by Chronic Granulomatous Disease (CDG), a systemic disorder caused by a genetic deficiency in NADPH oxidase and consequent impairment in NET formation. Initially described as “a fatal granulomatous disease of childhood’’, CGD used to be associated with recurrent respiratory infections and high mortality rate before the introduction of effective antimicrobial treatments (77). The protective role of NETs, particularly in the early phase of infection, in controlling pathogen growth and dissemination is also evident from experimental observations (78–84). In a mouse model of polymicrobial sepsis by cecal ligation puncture (CLP), Czaikoski and coworkers report that DNase treatment increased systemic bacterial burden and decreased survival (78). Consistent results were also reported by Meng and coworkers that, using DNase treatment in a similar mouse model of sepsis, described a profound and sustainable reduction of NET-mediated bactericidal activity associated with worst inflammation, more severe organ injury and higher early mortality (79). Finally, better outcomes in sepsis were reported by Lee and coworkers in mice deficient for phospholipase D2 (PLD2) that exhibit an intrinsic up-regulation of PAD4 activity associated with augmenting NET formation and bacterial killing (80).

Although the contribution of NETs in pathogen clearance is well established, aberrant NET release, particularly into the lung, has also been associated with the exacerbation of inflammation, immunothrombosis, and tissue injury (85–90). For example, NETs formed during LPS-induced ALI have been shown to directly cause organ damage and exacerbate an inflammatory response characterized by leukocyte accumulation, diffuse alveolar damage and cytokine release which could be improved by DNase treatment (85). Further details into the mechanism of NeT-mediated damage during bacterial sepsis were provided by McDonald and coworkers that recently described the implication of NET–platelet–thrombin axis in the promotion of intravascular coagulation and showed that PAD4 deficient mice or mice that received DNase have reduced microvascular occlusion and organ dysfunction (86). Blocking NET release by pharmacological inhibition of Gasdermin D (91) or PAD4 (41, 92) similarly reduced signs of sepsis-related multi-organ dysfunction and ALI. Moreover, data on critically ill subjects affected with infectious pneumonia and sepsis indicate that higher circulating NETs are associated with the worst clinical outcomes and directly correlate with organ dysfunction (12, 69, 93–96). Finally, a meta-analysis of randomized controlled trials on the use of Sivelestat for the treatment of ARDS in subjects with sepsis showed improved lung function even in the absence of a significant increased overall survival (97).

A possible explanation for the complex interplay between antimicrobial and pro-inflammatory NET function has been recently proposed by Lafrançais and coworkers (95). In this study, the authors confirmed the presence of higher levels of NETs in ARDS subjects with infectious etiology that correlated with the worst clinical outcomes. Moreover, in a mouse model of bacterial pneumonia, they found that NET release exacerbated signs of ALI, which could be attenuated in PAD4 deficient mice or by DNase administration. Interestingly, increased bacterial growth, particularly observed in mice with a genetic defect in NETosis, partially counteracted these beneficial effects, suggesting that the early release of NETs is important in initially control the infection and that ARDS in sepsis may represent the maladaptive consequence of this primarily defense mechanism (95). This view is consistent with previous findings showing that early DNase administration in a CLP model of sepsis increased signs of ALI but that the same treatment delivered in later phases was beneficial in reducing the level of systemic inflammation and increased survival (98).

In conclusion, the experimental evidence summarized in **Table 1**, and the clinical observations from sepsis subjects with ARDS, indicate that NETs play a dual role in ALI associated with sepsis and pneumonia. On the one hand, the early release of NETs at the site of infection is essential for controlling pathogen growth, and defects in NETosis or early NET degradation are associated with recurrent pneumonia and disseminated infections. However, NET exuberant release, particularly in a later phase dominated by post-infectious inflammatory events, appears largely detrimental and concurrently exacerbates tissue damage and ALI. Possible therapeutic strategies directed to modulate NET-mediated lung damage during infection should consider this dynamic relation between the early contribution to pathogen control and the late post-infectious exacerbation of systemic inflammation.

**Table 1:** The effects of NET treatment in experimental models of sepsis.

NET treatment	Sepsis model	Microbial Burden	Tissue Injury	Survival	Reference
DNase (NET digestion)	CLP	Increased	Increased	Reduced	Czaikoski et al. (78) Meng et al. (79)
DNase (NET digestion)	E.Coli IP infection	Increased	Decreased	–	McDonald et al. (84)
DNase (NET digestion)	MRSA IT infection	No effect	Decreased	Increased	Lafrancais et al. (95)
PAD4-/- (NET inhibition)	MRSA IT infection	Increased	Decreased	No effect	Lafrancais et al. (95)
Alcohol intoxication (NET inhibition)	CLP	Increased	–	Reduced	Jin et al. (82)
DNase (NET digestion) + Antibiotics	CLP	Reduced	Reduced	Increased	Czaikoski et al. (78)
Delayed DNase (NET digestion)	CLP	Reduced	Reduced	Increased	Mai et al. (98)
Disulfiram (NET inhibition)	CLP	–	Reduced	Increased	Silva et al. (91)
Cl-Amidine (NET inhibition)	CLP	–	–	Increased	Biron et al. (41)
DNase (NET digestion)	LPS	–	Reduced	Increased	Czaikoski et al. (78)
DNase (NET digestion)	LPS	–	Reduced	–	Liu et al. (85)

- , ‘Not Studied’ ; CLP, Cecal Ligation Puncture; LPS, lipopolysaccharide; MRSA, Methicillin-resistant Staphilococcus aureus; E.coli, Escherichia Coli; PAD4, Peptidylarginine deiminase 4; IP, intra-peritoneal; IT, intra-tracheal.

### COVID-19

COVID-19 is a global pandemic caused by severe acute respiratory syndrome coronavirus-2 (SARS-CoV-2) infection, which in its most severe clinical presentation, is associated with ALI/ARDS and multiple organ failure (99–101). COVID-19 infection with a high neutrophil-to-lymphocyte ratio is generally associated with the worst clinical outcomes (102, 103). Neutrophil activation signature is a prominent feature of circulating leukocyte transcriptomes of severe cases (104, 105). Additionally, single-cell RNA sequencing (scRNA-seq) analysis of COVID-19 whole blood samples has revealed distinct neutrophil clusters associated with NET release and disease severity (106). In order to characterize COVID-19 immunopathogenesis, several studies have focused on neutrophil effector functions, and in particular, NETosis (107–111). SARS-CoV-2 infection has been shown to directly induce NETosis in healthy neutrophils (111). NETosis has also been reported as a predictor of COVID-19 severity (110, 112). Post-mortem examinations of lungs from COVID-19 patients reveal diffuse neutrophil infiltration with abundant NET deposition frequently associated with platelet accumulation and microvascular thrombosis (113–116). This inflammatory pattern of thrombosis is consistent with findings from Zuo and colleagues, who showed a positive correlation between markedly elevated cell-free DNA and D-dimer levels, a degradation product of fibrin (110). The pathogenic role of NET release has also been described in different thrombosis-mediated inflammatory states associated with ARDS (90, 117). One current view of the underlying causes of ALI-mediated pulmonary thrombosis is that NET web-like structures immobilize and activate platelets and leukocytes. Through mechanisms that have yet to be fully elucidated, this induces a feed-forward loop that triggers additional intravascular NETosis and higher local concentrations of histones and proteases that increase endothelial permeability and promote microvascular obstruction (117). Given the prominent role of immunothrombosis in the pathogenesis of COVID-19 ALI/ARDS, pharmacological NET inhibition or degradation could represent an intriguing approach to attenuate disease severity and improve survival. A recent study by Fisher and colleagues supports this notion by showing remarkable improvement in clinical outcomes in five severely ill COVID-19 subjects treated with off-label aerosolized recombinant human (rh) DNase (118). Similar findings were also observed in different single-center case series and cohort studies where nebulized rhDNase administration showed favorable clinical outcomes in the absence of drug-associated toxicities (119, 120). Although the results from these small observational studies are encouraging, larger and randomized clinical trials will be required to assess the long terms effects of DNase treatment on COVID-19 ARDS.

### Transfusion-related acute lung injury (TRALI)

TRALI is a form of ALI that occurs within six hours of transfusion that cannot be explained by another ALI risk factor (121). Neutrophil activation and sequestration within the pulmonary capillaries in response to blood components is considered a central feature in the pathogenesis of TRALI (122). Recent insights into the mechanisms of neutrophil-mediated tissue damage indicate that NETs form in the lung and contribute to the pathogenesis of this disease (123–125). Using an established mouse model of TRALI (126), Caudrillier and coworkers show that platelet sequestration increases with NET proximity and that aspirin treatment decreases NET formation and NET-associated platelets (123). Moreover, human platelets activated with the PAR-1 agonist, thrombin receptor-activating peptide (TRAP), induced robust NET release *via* Thromboxane A2 (123). Further investigation into how platelets drive NETosis revealed dependence on the canonical Raf/MEK/ERK signaling pathway that is also critical to PMA-induced NADPH-dependent NETosis (127). Despite the reported association of NETs with TRALI, a different study by Thomas and colleagues found that platelet depletion did not completely abrogate NETosis and that FcγR engagement by anti-neutrophil antibodies was sufficient to promote NET release (124). Moreover, this study showed that DNase administration effectively prevented NET-mediated ALI only when delivered through inhalation, probably because of the predominant accumulation of NETs within alveolar spaces (124). NET inhibition rather than digestion also appears to be a promising approach to preventing TRALI as indicated by a recent study where the administration of Disulfiram, an FDA-approved gasdermin D inhibitor for the treatment of alcohol abuse, prevented signs of lung injury (125).

In summary, NETs appear to have a pathogenic role in TRALI, and preclinical observations indicate that NET inhibition or digestion may improve TRALI outcomes. However, conflicting reports as to the specific contribution of platelet activation to NET-mediated tissue injury in TRALI further highlight the need for studies to dissect the molecular mechanisms of NETosis *in vivo*.

### Lung ischemia-reperfusion injury (LIRI)

LIRI is a primarily sterile inflammatory disease occurring in lungs that sustain ischemic damage followed by reperfusion. LIRI is typically associated with pulmonary embolisms and various cardiothoracic surgical procedures, including lung transplantation (LTx) (128). The physiopathological mechanisms of LIRI are incredibly complex (129), and the specific contribution of neutrophils is still debated (130, 131). The prevailing view is that LIRI is a bimodal process, with an early phase primarily dependent on pulmonary macrophages and activated platelets followed by later neutrophil-mediated damage (132–134).

A recent study by Sayah and colleagues demonstrated a pathogenic role for NETs in primary graft dysfunction (PGD), a common form of LTx-related LIRI occurring within 72 hours. after surgery. Using a mouse model of syngeneic orthotopic left lung transplant (OLT), this group detected the presence of intra-graft NETs in association with platelet activation. Pretreatment with aspirin or intrabronchial administration of DNase were both able to attenuate signs of PGD in the OLT model (135). A later study by our group extended these findings by visualizing intragraft NETosis by intravital 2-photon microscopy in an allogeneic mouse model of OLT. Interestingly, the systemic administration of DNase improved lung function but at the cost of increasing alloimmune responses and graft rejection due to the release of inflammatory NET fragments. However, directly targeting NET generation by the genetic deletion or pharmacological inhibition of PAD4 prevented alloimmune responses and allograft rejection (136). The unintended consequences associated with NET digestion have been increasingly described in different animal models, raising the question of whether it is more effective to prevent NETosis in lieu of DNase treatment (137). For example, NETs have been described to merge in high-density structures named aggNETs, which are thought to prevent bystander tissue injury as well as contribute to wound healing and inflammation resolution through driving the proteolytic degradation of cytokines and histones (138–140). Most recently, our group has provided further insight into the mechanisms of NET-mediated graft injury by showing that necroptosis, occurring in response to warm-ischemia, drives early neutrophil accumulation into the sub-pleural capillary network resulting in NET-mediated microvascular damage (59). Necroptosis is a Receptor Interacting Serine/Threonine Kinase 3 activation-mediated form of cell death that leads to the extracellular release of various alarmins, including mitochondrial-derived DAMPs (mtDAMPs) (141). Additionally, our group and others have shown that LIRI leads to the release of mtDAMPs, which guide neutrophil intragraft trafficking, stimulate ROS production and trigger NETosis (61, 142).

Altogether, the observations from the LIRI models and human LTx recipients with PGD support the notion that DAMPs released from the ischemic tissues induce innate immune responses resulting in NETosis and tissue injury. Interfering with the mechanisms that activate NETosis rather than enhancing the degradation of NETs appears a more desirable option for attenuating LTx-related ALI without increasing the risk of allograft rejection.

### Ventilator-induced lung injury (VILI)

VILI is a form of ALI triggered by mechanical ventilation (143). Neutrophil recruitment in response to VILI is driven by L-selectin expression and is promoted by stretch-induced inflammatory events (144) that lead to the release of neutrophil chemokines such as CXCL1 (KC) and CXCL2/3 (MIP-2) (145). Short periods of mechanical ventilation have been shown to be associated with the release of pro-inflammatory cytokines, including TNF-α, IL-1β and IL-8 (146, 147), which are well-known activators of NETosis (148). Accordingly, recent studies have focused on the possible contribution of NETs to VILI pathogenesis. Using a “double-hit” model of intra-tracheal LPS challenge followed by high tidal mechanical ventilation, Yildiz and colleagues showed that VILI is associated with significant NET release. DNase, also administrated intratracheally, reduced BAL markers of NETs and improved lung compliance but did not affect other measures of lung injury including oxygenation, inflammatory cell infiltration and lung permeability (149). The singular improvement in lung compliance led the authors to hypothesize that DNase treatment decreased airway secretion viscosity rather than imparting VILI protection (149). In contrast to these findings, Rossaint and coworkers found that targeting NETs with DNase effectively protected mice from ALI in a single hit model of VILI (150). Moreover, the group further demonstrated that VILI-induced NETosis is a platelet-dependent process requiring simultaneous stimulation of integrins and G-protein–coupled receptors on neutrophils (150). Using the same single hit model of VILI, Li and coworkers confirmed the beneficial effect of DNase treatment in preventing ALI and showed that TLR4 signaling is implicated in the release of NETs in VILI (151).

In summary, the current evidence shows that NETs are released during VILI and that targeting NETs may be beneficial in preventing injury in this setting. The differing effects observed with DNase treatment may be related to the use of alternative experimental models suggesting further pre-clinical evaluation is likely necessary to better characterize the effects of NETosis on VILI.

### Trauma

Trauma is defined as an injury caused by an external physical force, as it may result in the consequence of blunt force impact, penetrating injuries, or extensive thermal and chemical burns. Although advancements in surgery and resuscitation have substantially increased the chance of surviving a major trauma, some patients still develop systemic inflammatory response syndrome (SIRS) and infections (152). ARDS is a well-known complication of major trauma, and it represents one of its leading causes of death (153). Trauma is commonly associated with the loss of natural barrier function, which places large demands on neutrophils to scavenge tissue debris and control the diffusion of pathogens. However, due to the large microvascular bed and the long transit time, the lung represents a preferred site for the accumulation of activated neutrophils after trauma (154). NET release has been demonstrated in several experimental models of trauma (63, 155–157), and elevated levels of circulating NET markers have been associated with the worst clinical outcomes in subjects developing trauma-related ARDS (50). Interestingly, ARDS resulting from trauma seems to be overall associated with better clinical outcomes when compared to other forms of ARDS, suggesting the presence of peculiar immunological features (158–161). In this regard, trauma is a known driver of emergency hematopoiesis leading to a high number of circulating immature neutrophils (162) with impaired effector functions (163, 164). In addition, trauma induces the expansion of specific neutrophil subsets that are either unresponsive or display immunosuppressive activity, increasing the risk of secondary infections (165–167). Recent work by Hazeldine and colleagues has suggested that reduced neutrophil activity in the trauma setting is related to their ability to form NETs (168). This group demonstrated that a few minutes after the injury, neutrophils exhibit an activated phenotype characterized by enhanced glucose metabolism and NETosis. However, hours later, neutrophils became unresponsive, and their ability to form NETs dramatically decreased. To explain this apparent change in neutrophil function, the authors hypothesize that mtDAMPs, such as N-formylated peptides, desensitize neutrophils to further stimulation, effectively reducing their ability to release NETs (168). Recent findings from Itagaki and colleagues support the existence of this regulatory pathway. They showed that blocking the N-formylated peptide receptor-1 (FPR1) preserves neutrophil function after trauma (169). Other evidence, however, point to the pro-inflammatory role of mtDAMPs in activating neutrophils (58, 170, 171), increasing NETosis (61, 62), and predisposing to the development of SIRS (172). Further complicating this picture, studies of trauma subjects show reduced endogenous DNase activity and increased circulating cell-free DNA levels associated with post-injury complications (164, 173, 174). Despite the general state of immune unresponsiveness and the increased risk of infection, these studies suggest that restoring DNase activity is likely beneficial after trauma.

In summary, ALI associated with trauma appears to display peculiar immunological features, possibly related to the sudden and massive release of DAMPs and the alteration of regulatory mechanisms that promote DAMP clearance. How different DAMPs control neutrophil metabolism, differentiation and NETosis as well as shape host responses to trauma remains to be determined.

## Discussion

Increasing evidence suggests that NETs play an essential role in infectious and sterile ALI and that circulating NET markers can predict worst clinical outcomes in ARDS subjects (5). Most of the current literature has focused on the use of DNase in preventing NET-mediated ALI (175). However, although DNase has shown promising results in reducing signs of lung injury and inflammation associated with NET release, several questions about its global use to treat ARDS conditions remain. For example, does DNase-mediated NET degradation increase the risk of infection? In addition, do the rapid release of histones and proteases following DNAse treatment have unintended effects on outcomes? Preventing formation rather than digesting NETs may offer a more suitable alternative strategy to treat some forms of ALI. In this regard, selective inhibitors have been developed to prevent the generation and release of NETs at different stages of NETosis (17, 37). Nevertheless, it remains to be determined if NET formation inhibitors will be effective for most forms of ALI and, like DNase, could potentially dysregulate host defense. It also remains to be determined if NET inhibitors will be effective for all forms of NETosis, given the apparent redundancy in the pathways that generate NETs. Platelet activation and coagulation factors appear to be highly interconnected with NET release leading to a state of thromboinflammation (176). Although this mechanism appears to be extremely relevant in the ARDS associated with COVID-19, its contribution still needs to be precisely characterized for other ALI etiologies. In addition, the molecular details that control the crosstalk between innate immunity and coagulation remain poorly understood. DAMPs, particularly those derived from mitochondria, appear to be strongly involved in the activation of NETosis (61, 62). Interfering with their signals may represent a promising strategy to reduce exuberant NET release and prevent neutrophil desensitization to microbial PAMPs. However, to safely leverage these observations, pathways that control the delicate balance between neutrophil activation and desensitization will need further investigation.

In conclusion, future studies that better define the mechanisms involved in the formation, release, and degradation of NETs could form the basis of therapeutic strategies that target ALI without disabling pathogen surveillance.

## Author contributions

DS, FL, AK, DK, and AG contributed to conception and design of the study. DS wrote the first draft of the manuscript. All authors contributed to manuscript revision, read, and approved the submitted version.

## Funding

This study was funded by the NIH (NIH R01HL094601, NIH P01AI11650, NIH R01HL157407).

## Conflict of interest

The authors declare that the research was conducted in the absence of any commercial or financial relationships that could be construed as a potential conflict of interest.

## Publisher’s note

All claims expressed in this article are solely those of the authors and do not necessarily represent those of their affiliated organizations, or those of the publisher, the editors and the reviewers. Any product that may be evaluated in this article, or claim that may be made by its manufacturer, is not guaranteed or endorsed by the publisher.

## References

[1] Kulkarni HS Lee JS Bastarache JA Kuebler WM Downey GP Albaiceta GM. Update on the features and measurements of experimental acute lung injury in animals: An official American thoracic society workshop report. Am J Respir Cell Mol Biol (2022) 66:e1–e14. doi: 10.1165/rcmb.2021-0531ST 35103557 PMC8845128

[2] Millar FR Summers C Griffiths MJ Toshner MR Proudfoot AG. The pulmonary endothelium in acute respiratory distress syndrome: Insights and therapeutic opportunities. Thorax (2016) 71:462–73. doi: 10.1136/thoraxjnl-2015-207461 26968969

[3] Fanelli V Vlachou A Ghannadian S Simonetti U Slutsky AS Zhang H. Acute respiratory distress syndrome: New definition, current and future therapeutic options. J Thorac Dis (2013) 5:326–34. doi: 10.3978/j.issn.2072-1439.2013.04.05 PMC3698298 23825769

[4] Hendrickson KW Peltan ID Brown SM. The epidemiology of acute respiratory distress syndrome before and after coronavirus disease 2019. Crit Care Clin (2021) 37:703–16. doi: 10.1016/j.ccc.2021.05.001 PMC8449138 34548129

[5] Yang S-C Tsai Y-F Pan Y-L Hwang T-L. Understanding the role of neutrophils in acute respiratory distress syndrome. BioMed J (2021) 44:439–46. doi: 10.1016/j.bj.2020.09.001 PMC7481802 33087299

[6] Neubert E Meyer D Rocca F Günay G Kwaczala-Tessmann A Grandke J. Chromatin swelling drives neutrophil extracellular trap release. Nat Commun (2018) 9:3767. doi: 10.1038/s41467-018-06263-5 30218080 PMC6138659

[7] Yousefi S Mihalache C Kozlowski E Schmid I Simon HU. Viable neutrophils release mitochondrial DNA to form neutrophil extracellular traps. Cell Death Differ (2009) 16:1438–44. doi: 10.1038/cdd.2009.96 19609275

[8] McIlroy DJ Jarnicki AG Au GG Lott N Smith DW Hansbro PM. Mitochondrial DNA neutrophil extracellular traps are formed after trauma and subsequent surgery. J Crit Care (2014) 29:1133.e1–5. doi: 10.1016/j.jcrc.2014.07.013 25128442

[9] Lood C Blanco LP Purmalek MM Carmona-Rivera C De Ravin SS Smith CK. Neutrophil extracellular traps enriched in oxidized mitochondrial DNA are interferogenic and contribute to lupus-like disease. Nat Med (2016) 22:146–53. doi: 10.1038/nm.4027 PMC4742415 26779811

[10] Urban CF Ermert D Schmid M Abu-Abed U Goosmann C Nacken W. Neutrophil extracellular traps contain calprotectin, a cytosolic protein complex involved in host defense against candida albicans. PloS Pathog (2009) 5:e1000639. doi: 10.1371/journal.ppat.1000639 19876394 PMC2763347

[11] Papayannopoulos V. Neutrophil extracellular traps in immunity and disease. Nat Rev Immunol (2018) 18:134–47. doi: 10.1038/nri.2017.105 28990587

[12] Mikacenic C Moore R Dmyterko V West TE Altemeier WA Liles WC. Neutrophil extracellular traps (NETs) are increased in the alveolar spaces of patients with ventilator-associated pneumonia. Crit Care (2018) 22:358. doi: 10.1186/s13054-018-2290-8 30587204 PMC6307268

[13] Pan B Alam HB Chong W Mobley J Liu B Deng Q. CitH3: A reliable blood biomarker for diagnosis and treatment of endotoxic shock. Sci Rep (2017) 7:8972. doi: 10.1038/s41598-017-09337-4 28827548 PMC5567134

[14] Brinkmann V Reichard U Goosmann C Fauler B Uhlemann Y Weiss DS. Neutrophil extracellular traps kill bacteria. Science (2004) 303:1532–5. doi: 10.1126/science.1092385 15001782

[15] Fuchs TA Abed U Goosmann C Hurwitz R Schulze I Wahn V. Novel cell death program leads to neutrophil extracellular traps. J Cell Biol (2007) 176:231–41. doi: 10.1083/jcb.200606027 PMC2063942 17210947

[16] Remijsen Q Kuijpers TW Wirawan E Lippens S Vandenabeele P Vanden Berghe T. Dying for a cause: NETosis, mechanisms behind an antimicrobial cell death modality. Cell Death Differ (2011) 18:581–8. doi: 10.1038/cdd.2011.1 PMC3131909 21293492

[17] Sollberger G Choidas A Burn GL Habenberger P Di Lucrezia R Kordes S. Gasdermin d plays a vital role in the generation of neutrophil extracellular traps. Sci Immunol (2018) 3:eaar6689. doi: 10.1126/sciimmunol.aar6689 30143555

[18] Pilsczek FH Salina D Poon KKH Fahey C Yipp BG Sibley CD. A novel mechanism of rapid nuclear neutrophil extracellular trap formation in response to staphylococcus aureus. J Immunol (2010) 185:7413–25. doi: 10.4049/jimmunol.1000675 21098229

[19] Yipp BG Petri B Salina D Jenne CN Scott BNV Zbytnuik LD. Infection-induced NETosis is a dynamic process involving neutrophil multitasking. vivo Nat Med (2012) 18:1386–93. doi: 10.1038/nm.2847 PMC4529131 22922410

[20] Kenny EF Herzig A Krüger R Muth A Mondal S Thompson PR. Diverse stimuli engage different neutrophil extracellular trap pathways. eLife (2017) 6:e24437. doi: 10.7554/eLife.24437 28574339 PMC5496738

[21] Remijsen Q Vanden Berghe T Wirawan E Asselbergh B Parthoens E De Rycke R. Neutrophil extracellular trap cell death requires both autophagy and superoxide generation. Cell Res (2011) 21:290–304. doi: 10.1038/cr.2010.150 21060338 PMC3193439

[22] Desai J Kumar SV Mulay SR Konrad L Romoli S Schauer C. PMA and crystal-induced neutrophil extracellular trap formation involves RIPK1-RIPK3-MLKL signaling. Eur J Immunol (2016) 46:223–9. doi: 10.1002/eji.201545605 26531064

[23] Kaplan MJ Radic M. Neutrophil extracellular traps (NETs): Double-edged swords of innate immunity. J Immunol (2012) 189:2689–95. doi: 10.4049/jimmunol.1201719 PMC3439169 22956760

[24] Stapels DAC Geisbrecht BV Rooijakkers SHM. Neutrophil serine proteases in antibacterial defense. Curr Opin Microbiol (2015) 0:42–8. doi: 10.1016/j.mib.2014.11.002 PMC4323955 25461571

[25] Pham CTN. Neutrophil serine proteases fine-tune the inflammatory response. Int J Biochem Cell Biol (2008) 40:1317–33. doi: 10.1016/j.biocel.2007.11.008 PMC2440796 18180196

[26] O’Donoghue AJ Jin Y Knudsen GM Perera NC Jenne DE Murphy JE. Global substrate profiling of proteases in human neutrophil extracellular traps reveals consensus motif predominantly contributed by elastase. PloS One (2013) 8:e75141. doi: 10.1371/journal.pone.0075141 24073241 PMC3779220

[27] Carden D Xiao F Moak C Willis BH Robinson-Jackson S Alexander S. Neutrophil elastase promotes lung microvascular injury and proteolysis of endothelial cadherins. Am J Physiology-Heart Circulatory Physiol (1998) 275:H385–92. doi: 10.1152/ajpheart.1998.275.2.H385 9683424

[28] Hermant B Bibert S Concord E Dublet B Weidenhaupt M Vernet T. Identification of proteases involved in the proteolysis of vascular endothelium cadherin during neutrophil transmigration. J Biol Chem (2003) 278:14002–12. doi: 10.1074/jbc.M300351200 12584200

[29] Suttorp N Nolte A Wilke A Drenckhahn D. Human neutrophil elastase increases permeability of cultured pulmonary endothelial cell monolayers. Int J Microcirc Clin Exp (1993) 13:187–203. 8125708

[30] Suzuki K Okada H Takemura G Takada C Kuroda A Yano H. Neutrophil elastase damages the pulmonary endothelial glycocalyx in lipopolysaccharide-induced experimental endotoxemia. Am J Pathol (2019) 189:1526–35. doi: 10.1016/j.ajpath.2019.05.002 31108101

[31] Okeke EB Louttit C Fry C Najafabadi AH Han K Nemzek J. Inhibition of neutrophil elastase prevents neutrophil extracellular trap formation and rescues mice from endotoxic shock. Biomaterials (2020) 238:119836. doi: 10.1016/j.biomaterials.2020.119836 32045782 PMC7075277

[32] Papayannopoulos V Metzler KD Hakkim A Zychlinsky A. Neutrophil elastase and myeloperoxidase regulate the formation of neutrophil extracellular traps. J Cell Biol (2010) 191:677–91. doi: 10.1083/jcb.201006052 PMC3003309 20974816

[33] Martinod K Witsch T Farley K Gallant M Remold-O’Donnell E Wagner DD. Neutrophil elastase-deficient mice form neutrophil extracellular traps in an experimental model of deep vein thrombosis. J Thromb Haemostasis (2016) 14:551–8. doi: 10.1111/jth.13239 PMC4785059 26712312

[34] Tokuhiro T Ishikawa A Sato H Takita S Yoshikawa A Anzai R. Oxidized phospholipids and neutrophil elastase coordinately play critical roles in NET formation. Front Cell Dev Biol (2021) 9:718586. doi: 10.3389/fcell.2021.718586 34568331 PMC8458647

[35] Clancy DM Sullivan GP Moran HBT Henry CM Reeves EP McElvaney NG. Extracellular neutrophil proteases are efficient regulators of IL-1, IL-33, and IL-36 cytokine activity but poor effectors of microbial killing. Cell Rep (2018) 22:2937–50. doi: 10.1016/j.celrep.2018.02.062 29539422

[36] Hoeksema M van Eijk M Haagsman HP Hartshorn KL. Histones as mediators of host defense, inflammation and thrombosis. Future Microbiol (2016) 11:441–53. doi: 10.2217/fmb.15.151 PMC5549641 26939619

[37] Rohrbach A Slade D Thompson P Mowen K. Activation of PAD4 in NET formation. Front Immunol (2012) 3:360. doi: 10.3389/fimmu.2012.00360 23264775 PMC3525017

[38] Li P Li M Lindberg MR Kennett MJ Xiong N Wang Y. PAD4 is essential for antibacterial innate immunity mediated by neutrophil extracellular traps. J Exp Med (2010) 207:1853–62. doi: 10.1084/jem.20100239 PMC2931169 20733033

[39] Neeli I Khan SN Radic M. Histone deimination as a response to inflammatory stimuli in neutrophils. J Immunol (2008) 180:1895–902. doi: 10.4049/jimmunol.180.3.1895 18209087

[40] Tatsiy O McDonald PP. Physiological stimuli induce PAD4-dependent, ROS-independent NETosis, with early and late events controlled by discrete signaling pathways. Front Immunol (2018) 9:2036. doi: 10.3389/fimmu.2018.02036 30279690 PMC6153332

[41] Biron BM Chung C-S O’Brien XM Chen Y Reichner JS Ayala A. Cl-amidine prevents histone 3 citrullination and neutrophil extracellular trap formation, and improves survival in a murine sepsis model. J Innate Immun (2017) 9:22–32. doi: 10.1159/000448808 27622642 PMC5219946

[42] Zhan Y Ling Y Deng Q Qiu Y Shen J Lai H. HMGB1-mediated neutrophil extracellular trap formation exacerbates intestinal Ischemia/Reperfusion-induced acute lung injury. J Immunol (2022) 208:968–78. doi: 10.4049/jimmunol.2100593 35063996

[43] Guiducci E Lemberg C Küng N Schraner E Theocharides APA LeibundGut-Landmann S. Candida albicans-induced NETosis is independent of peptidylarginine deiminase 4. Front Immunol (2018) 9:1573. doi: 10.3389/fimmu.2018.01573 30038623 PMC6046457

[44] Claushuis TAM van der Donk LEH Luitse AL van Veen HA van der Wel NN van Vught LA. Role of peptidylarginine deiminase 4 in neutrophil extracellular trap formation and host defense during klebsiella pneumoniae–induced pneumonia-derived sepsis. J Immunol (2018) 201:1241–52. doi: 10.4049/jimmunol.1800314 29987161

[45] Warnatsch A Ioannou M Wang Q Papayannopoulos V. Neutrophil extracellular traps license macrophages and Th17 cells for cytokine production in atherosclerosis. Science (2015) 349:316–20. doi: 10.1126/science.aaa8064 PMC4854322 26185250

[46] Silk E Zhao H Weng H Ma D. The role of extracellular histone in organ injury. Cell Death Dis (2017) 8:e2812–2. doi: 10.1038/cddis.2017.52 PMC5520745 28542146

[47] Semeraro F Ammollo CT Morrissey JH Dale GL Friese P Esmon NL. Extracellular histones promote thrombin generation through platelet-dependent mechanisms: Involvement of platelet TLR2 and TLR4. Blood (2011) 118:1952–61. doi: 10.1182/blood-2011-03-343061 PMC3158722 21673343

[48] Fuchs TA Bhandari AA Wagner DD. Histones induce rapid and profound thrombocytopenia in mice. Blood (2011) 118:3708–14. doi: 10.1182/blood-2011-01-332676 PMC3186342 21700775

[49] Huang H Chen H-W Evankovich J Yan W Rosborough BR Nace GW. Histones activate the NLRP3 inflammasome in kupffer cells during sterile inflammatory liver injury. J Immunol (2013) 191:2665–79. doi: 10.4049/jimmunol.1202733 PMC3777242 23904166

[50] Abrams ST Zhang N Manson J Liu T Dart C Baluwa F. Circulating histones are mediators of trauma-associated lung injury. Am J Respir Crit Care Med (2013) 187:160–9. doi: 10.1164/rccm.201206-1037OC PMC3570656 23220920

[51] Saffarzadeh M Juenemann C Queisser MA Lochnit G Barreto G Galuska SP. Neutrophil extracellular traps directly induce epithelial and endothelial cell death: A predominant role of histones. PloS One (2012) 7:e32366. doi: 10.1371/journal.pone.0032366 22389696 PMC3289648

[52] Kolaczkowska E Jenne CN Surewaard BGJ Thanabalasuriar A Lee W-Y Sanz M-J. Molecular mechanisms of NET formation and degradation revealed by intravital imaging in the liver vasculature. Nat Commun (2015) 6:6673. doi: 10.1038/ncomms7673 25809117 PMC4389265

[53] Fuchs TA Brill A Duerschmied D Schatzberg D Monestier M Myers DD. Extracellular DNA traps promote thrombosis. Proc Natl Acad Sci U.S.A. (2010) 107:15880–5. doi: 10.1073/pnas.1005743107 PMC2936604 20798043

[54] Gould TJ Vu TT Swystun LL Dwivedi DJ Mai SHC Weitz JI. Neutrophil extracellular traps promote thrombin generation through platelet-dependent and platelet-independent mechanisms. Arterioscler Thromb Vasc Biol (2014) 34:1977–84. doi: 10.1161/ATVBAHA.114.304114 25012129

[55] Yang J Wu Z Long Q Huang J Hong T Liu W. Insights into immunothrombosis: The interplay among neutrophil extracellular trap, Von willebrand factor, and ADAMTS13. Front Immunol (2020) 11:610696. doi: 10.3389/fimmu.2020.610696 33343584 PMC7738460

[56] Taanman J-W. The mitochondrial genome: Structure, transcription, translation and replication. Biochim Biophys Acta (BBA) - Bioenergetics (1999) 1410:103–23. doi: 10.1016/S0005-2728(98)00161-3 10076021

[57] Gray MW. Mitochondrial evolution. Cold Spring Harb Perspect Biol (2012) 4:a011403. doi: 10.1101/cshperspect.a011403 22952398 PMC3428767

[58] Zhang Q Raoof M Chen Y Sumi Y Sursal T Junger W. Circulating mitochondrial DAMPs cause inflammatory responses to injury. Nature (2010) 464:104–7. doi: 10.1038/nature08780 PMC2843437 20203610

[59] Zhang Q Itagaki K Hauser CJ. Mitochondrial DNA is released by shock and activates neutrophils *via* P38 map kinase. Shock (2010) 34:55–9. doi: 10.1097/SHK.0b013e3181cd8c08 19997055

[60] Shimada K Crother TR Karlin J Dagvadorj J Chiba N Chen S. Oxidized mitochondrial DNA activates the NLRP3 inflammasome during apoptosis. Immunity (2012) 36:401–14. doi: 10.1016/j.immuni.2012.01.009 PMC3312986 22342844

[61] Mallavia B Liu F Lefrançais E Cleary SJ Kwaan N Tian JJ. Mitochondrial DNA stimulates TLR9-dependent neutrophil extracellular trap formation in primary graft dysfunction. Am J Respir Cell Mol Biol (2020) 62:364–72. doi: 10.1165/rcmb.2019-0140OC PMC7055700 31647878

[62] Itagaki K Kaczmarek E Lee YT Tang IT Isal B Adibnia Y. Mitochondrial DNA released by trauma induces neutrophil extracellular traps. PloS One (2015) 10:e0120549. doi: 10.1371/journal.pone.0120549 25774524 PMC4361684

[63] Liu L Mao Y Xu B Zhang X Fang C Ma Y. Induction of neutrophil extracellular traps during tissue injury: Involvement of STING and toll-like receptor 9 pathways. Cell Prolif (2019) 52:e12579. doi: 10.1111/cpr.12579 30851061 PMC6536408

[64] Singer M Deutschman CS Seymour CW Shankar-Hari M Annane D Bauer M. The third international consensus definitions for sepsis and septic shock (Sepsis-3). JAMA (2016) 315:801–10. doi: 10.1001/jama.2016.0287 PMC4968574 26903338

[65] Hu Q Hao C Tang S. From sepsis to acute respiratory distress syndrome (ARDS): Emerging preventive strategies based on molecular and genetic researches. Biosci Rep (2020) 40:BSR20200830. doi: 10.1042/BSR20200830 32319516 PMC7199454

[66] Rhee C Jones TM Hamad Y Pande A Varon J O’Brien C. Prevalence, underlying causes, and preventability of sepsis-associated mortality in US acute care hospitals. JAMA Network Open (2019) 2:e187571. doi: 10.1001/jamanetworkopen.2018.7571 30768188 PMC6484603

[67] Lee K-Y. Pneumonia, acute respiratory distress syndrome, and early immune-modulator therapy. Int J Mol Sci (2017) 18:388. doi: 10.3390/ijms18020388 PMC5343923 28208675

[68] De Freitas Caires N Gaudet A Portier L Tsicopoulos A Mathieu D Lassalle P. Endocan, sepsis, pneumonia, and acute respiratory distress syndrome. Crit Care (2018) 22:280. doi: 10.1186/s13054-018-2222-7 30367649 PMC6204032

[69] Denning N-L Aziz M Gurien SD Wang P. DAMPs and NETs in sepsis. Front Immunol (2019) 10:2536. doi: 10.3389/fimmu.2019.02536 31736963 PMC6831555

[70] Kang J-W Kim S-J Cho H-I Lee S-M. DAMPs activating innate immune responses in sepsis. Ageing Res Rev (2015) 24:54–65. doi: 10.1016/j.arr.2015.03.003 25816752

[71] Raymond SL Holden DC Mira JC Stortz JA Loftus TJ Mohr AM. Microbial recognition and danger signals in sepsis and trauma. Biochim Biophys Acta (BBA) - Mol Basis Dis (2017) 1863:2564–73. doi: 10.1016/j.bbadis.2017.01.013 PMC5519458 28115287

[72] O’Brien XM Biron BM Reichner JS. Consequences of extracellular trap formation in sepsis. Curr Opin Hematol (2017) 24:66–71. doi: 10.1097/MOH.0000000000000303 27820735 PMC5892420

[73] Azzouz L Cherry A Riedl M Khan M Pluthero FG Kahr WHA. Relative antibacterial functions of complement and NETs: NETs trap and complement effectively kills bacteria. Mol Immunol (2018) 97:71–81. doi: 10.1016/j.molimm.2018.02.019 29571059

[74] Storisteanu DML Pocock JM Cowburn AS Juss JK Nadesalingam A Nizet V. Evasion of neutrophil extracellular traps by respiratory pathogens. Am J Respir Cell Mol Biol (2017) 56:423–31. doi: 10.1165/rcmb.2016-0193PS PMC5449512 27854516

[75] Beiter K Wartha F Albiger B Normark S Zychlinsky A Henriques-Normark B. An endonuclease allows streptococcus pneumoniae to escape from neutrophil extracellular traps. Curr Biol (2006) 16:401–7. doi: 10.1016/j.cub.2006.01.056 16488875

[76] Eby JC Gray MC Hewlett EL. Cyclic AMP-mediated suppression of neutrophil extracellular trap formation and apoptosis by the bordetella pertussis adenylate cyclase toxin. Infect Immun (2014) 82:5256–69. doi: 10.1128/IAI.02487-14 PMC4249293 25287922

[77] Arnold DE Heimall JR. A review of chronic granulomatous disease. Adv Ther (2017) 34:2543–57. doi: 10.1007/s12325-017-0636-2 PMC5709447 29168144

[78] Czaikoski PG Mota JMSC Nascimento DC Sônego F Castanheira FV Melo PH. Neutrophil extracellular traps induce organ damage during experimental and clinical sepsis. PloS One (2016) 11:e0148142. doi: 10.1371/journal.pone.0148142 26849138 PMC4743982

[79] Meng W Paunel-Görgülü A Flohé S Hoffmann A Witte I MacKenzie C. Depletion of neutrophil extracellular traps *In vivo* results in hypersusceptibility to polymicrobial sepsis in mice. Crit Care (2012) 16:R137. doi: 10.1186/cc11442 22835277 PMC3580722

[80] Lee SK Kim SD Kook M Lee HY Ghim J Choi Y. Phospholipase D2 drives mortality in sepsis by inhibiting neutrophil extracellular trap formation and down-regulating CXCR2. J Exp Med (2015) 212:1381–90. doi: 10.1084/jem.20141813 PMC4548059 26282875

[81] Yost CC Cody MJ Harris ES Thornton NL McInturff AM Martinez ML. Impaired neutrophil extracellular trap (NET) formation: A novel innate immune deficiency of human neonates. Blood (2009) 113:6419–27. doi: 10.1182/blood-2008-07-171629 PMC2710935 19221037

[82] Jin L Batra S Jeyaseelan S. Diminished neutrophil extracellular trap (NET) formation is a novel innate immune deficiency induced by acute ethanol exposure in polymicrobial sepsis, which can be rescued by CXCL1. PloS Pathog (2017) 13:e1006637. doi: 10.1371/journal.ppat.1006637 28922428 PMC5626520

[83] Clark SR Ma AC Tavener SA McDonald B Goodarzi Z Kelly MM. Platelet TLR4 activates neutrophil extracellular traps to ensnare bacteria in septic blood. Nat Med (2007) 13:463–9. doi: 10.1038/nm1565 17384648

[84] McDonald B Urrutia R Yipp BG Jenne CN Kubes P. Intravascular neutrophil extracellular traps capture bacteria from the bloodstream during sepsis. Cell Host Microbe (2012) 12:324–33. doi: 10.1016/j.chom.2012.06.011 22980329

[85] Liu S Su X Pan P Zhang L Hu Y Tan H. Neutrophil extracellular traps are indirectly triggered by lipopolysaccharide and contribute to acute lung injury. Sci Rep (2016) 6:37252. doi: 10.1038/srep37252 27849031 PMC5110961

[86] McDonald B Davis RP Kim S-J Tse M Esmon CT Kolaczkowska E. Platelets and neutrophil extracellular traps collaborate to promote intravascular coagulation during sepsis in mice. Blood (2017) 129:1357–67. doi: 10.1182/blood-2016-09-741298 PMC5345735 28073784

[87] Camicia G Pozner R de Larrañaga G. Neutrophil extracellular traps in sepsis. Shock (2014) 42:286–94. doi: 10.1097/SHK.0000000000000221 25004062

[88] Colón DF Wanderley CW Franchin M Silva CM Hiroki CH Castanheira FVS. Neutrophil extracellular traps (NETs) exacerbate severity of infant sepsis. Crit Care (2019) 23:113. doi: 10.1186/s13054-019-2407-8 30961634 PMC6454713

[89] Yang S Qi H Kan K Chen J Xie H Guo X. Neutrophil extracellular traps promote hypercoagulability in patients with sepsis. Shock (2017) 47:132–9. doi: 10.1097/SHK.0000000000000741 27617671

[90] Zhang H Zhou Y Qu M Yu Y Chen Z Zhu S. Tissue factor-enriched neutrophil extracellular traps promote immunothrombosis and disease progression in sepsis-induced lung injury. Front Cell Infect Microbiol (2021) 11:677902. doi: 10.3389/fcimb.2021.677902 34336711 PMC8317465

[91] Silva CMS Wanderley CWS Veras FP Sonego F Nascimento DC Gonçalves AV. Gasdermin d inhibition prevents multiple organ dysfunction during sepsis by blocking NET formation. Blood (2021) 138:2702–13. doi: 10.1182/blood.2021011525 PMC8703366 34407544

[92] Li Y Liu Z Liu B Zhao T Chong W Wang Y. Citrullinated histone H3 – a novel target for treatment of sepsis. Surgery (2014) 156:229–34. doi: 10.1016/j.surg.2014.04.009 PMC4267527 24957671

[93] Margraf S Lögters T Reipen J Altrichter J Scholz M Windolf J. Neutrophil-derived circulating free DNA (Cf-DNA/NETs): A potential prognostic marker for posttraumatic development of inflammatory second hit and sepsis. Shock (2008) 30:352–8. doi: 10.1097/SHK.0b013e31816a6bb1 18317404

[94] Maruchi Y Tsuda M Mori H Takenaka N Gocho T Huq MA. Plasma myeloperoxidase-conjugated DNA level predicts outcomes and organ dysfunction in patients with septic shock. Crit Care (2018) 22:176. doi: 10.1186/s13054-018-2109-7 30005596 PMC6045839

[95] Lefrançais E Mallavia B Zhuo H Calfee CS Looney MR. Maladaptive role of neutrophil extracellular traps in pathogen-induced lung injury. JCI Insight (2018) 3:98178. doi: 10.1172/jci.insight.98178 29415887 PMC5821185

[96] Ebrahimi F Giaglis S Hahn S Blum CA Baumgartner C Kutz A. Markers of neutrophil extracellular traps predict adverse outcome in community-acquired pneumonia: Secondary analysis of a randomised controlled trial. Eur Respir J (2018) 51:1701389. doi: 10.1183/13993003.01389-2017 29519921

[97] Pu S Wang D Liu D Zhao Y Qi D He J. Effect of sivelestat sodium in patients with acute lung injury or acute respiratory distress syndrome: A meta-analysis of randomized controlled trials. BMC Pulm Med (2017) 17:148. doi: 10.1186/s12890-017-0498-z 29162066 PMC5699178

[98] Mai SHC Khan M Dwivedi DJ Ross CA Zhou J Gould TJ. Delayed but not early treatment with DNase reduces organ damage and improves outcome in a murine model of sepsis. Shock (2015) 44:166–72. doi: 10.1097/SHK.0000000000000396 26009820

[99] Acosta MAT Singer BD. Pathogenesis of COVID-19-Induced ARDS: Implications for an ageing population. Eur Respir J (2020) 56(3):2002049. doi: 10.1183/13993003.02049-2020 32747391 PMC7397945

[100] Yuki K Fujiogi M Koutsogiannaki S. COVID-19 pathophysiology: A review. Clin Immunol (2020) 215:108427. doi: 10.1016/j.clim.2020.108427 32325252 PMC7169933

[101] Carsana L Sonzogni A Nasr A Rossi RS Pellegrinelli A Zerbi P. Pulmonary post-mortem findings in a series of COVID-19 cases from northern Italy: A two-centre descriptive study. Lancet Infect Dis (2020) 20:1135–40. doi: 10.1016/S1473-3099(20)30434-5 PMC7279758 32526193

[102] Li X Liu C Mao Z Xiao M Wang L Qi S. Predictive values of neutrophil-to-Lymphocyte ratio on disease severity and mortality in COVID-19 patients: A systematic review and meta-analysis. Crit Care (2020) 24:647. doi: 10.1186/s13054-020-03374-8 33198786 PMC7667659

[103] Liu J Liu Y Xiang P Pu L Xiong H Li C. Neutrophil-To-Lymphocyte ratio predicts critical illness patients with 2019 coronavirus disease in the early stage. J Trans Med (2020) 18:206. doi: 10.1186/s12967-020-02374-0 PMC7237880 32434518

[104] Meizlish ML Pine AB Bishai JD Goshua G Nadelmann ER Simonov M. A neutrophil activation signature predicts critical illness and mortality in COVID-19. Blood Adv (2021) 5:1164–77. doi: 10.1182/bloodadvances.2020003568 PMC7908851 33635335

[105] Aschenbrenner AC Mouktaroudi M Krämer B Oestreich M Antonakos N Nuesch-Germano M. Disease severity-specific neutrophil signatures in blood transcriptomes stratify COVID-19 patients. Genome Med (2021) 13:7. doi: 10.1186/s13073-020-00823-5 33441124 PMC7805430

[106] Schulte-Schrepping J Reusch N Paclik D Baßler K Schlickeiser S Zhang B. Severe COVID-19 is marked by a dysregulated myeloid cell compartment. Cell (2020) 182:1419–1440.e23. doi: 10.1016/j.cell.2020.08.001 32810438 PMC7405822

[107] Reusch N De Domenico E Bonaguro L Schulte-Schrepping J Baßler K Schultze JL. Neutrophils in COVID-19. Front Immunol (2021) 12:652470. doi: 10.3389/fimmu.2021.652470 33841435 PMC8027077

[108] Cavalcante-Silva LHA Carvalho DCM Lima É de A Galvão JGFM da Silva JS de F de Sales-Neto JM. Neutrophils and COVID-19: The road so far. Int Immunopharmacol (2021) 90:107233. doi: 10.1016/j.intimp.2020.107233 33290963 PMC7703515

[109] Arcanjo A Logullo J Menezes CCB de Souza Carvalho Giangiarulo TC dos Reis MC de Castro GMM. The emerging role of neutrophil extracellular traps in severe acute respiratory syndrome coronavirus 2 (COVID-19). Sci Rep (2020) 10:19630. doi: 10.1038/s41598-020-76781-0 33184506 PMC7665044

[110] Zuo Y Yalavarthi S Shi H Gockman K Zuo M Madison JA. Neutrophil extracellular traps in COVID-19. JCI Insight (2020) 5:138999. doi: 10.1172/jci.insight.138999 32329756 PMC7308057

[111] Veras FP Pontelli MC Silva CM Toller-Kawahisa JE de Lima M Nascimento DC. SARS-CoV-2–triggered neutrophil extracellular traps mediate COVID-19 pathologySARS-CoV-2 directly triggers ACE-dependent NETs. J Exp Med (2020) 217:e20201129. doi: 10.1084/jem.20201129 32926098 PMC7488868

[112] Janiuk K Jabłońska E Garley M. Significance of NETs formation in COVID-19. Cells (2021) 10:151. doi: 10.3390/cells10010151 33466589 PMC7828704

[113] Barnes BJ Adrover JM Baxter-Stoltzfus A Borczuk A Cools-Lartigue J Crawford JM. Targeting potential drivers of COVID-19: Neutrophil extracellular trapsneutrophil extracellular traps in COVID-19. J Exp Med (2020) 217:e20200652. doi: 10.1084/jem.20200652 32302401 PMC7161085

[114] Parra-Medina R Herrera S Mejia J. Systematic review of microthrombi in COVID-19 autopsies. Actq Haematol (2021), 144(5):476–83. doi: 10.1159/000515104 PMC8089413 33873184

[115] Fang X-Z Wang Y-X Xu J-Q He Y-J Peng Z-K Shang Y. Immunothrombosis in acute respiratory dysfunction of COVID-19. Front Immunol (2021) 12:651545. doi: 10.3389/fimmu.2021.651545 34149692 PMC8207198

[116] Middleton EA He X-Y Denorme F Campbell RA Ng D Salvatore SP. Neutrophil extracellular traps contribute to immunothrombosis in COVID-19 acute respiratory distress syndrome. Blood (2020) 136:1169–79. doi: 10.1182/blood.2020007008 PMC7472714 32597954

[117] Frantzeskaki F Armaganidis A Orfanos SE. Immunothrombosis in acute respiratory distress syndrome: Cross talks between inflammation and coagulation. RES (2017) 93:212–25. doi: 10.1159/000453002 27997925

[118] Fisher J Mohanty T Karlsson CAQ Khademi SMH Malmström E Frigyesi A. Proteome profiling of recombinant DNase therapy in reducing NETs and aiding recovery in COVID-19 patients. Mol Cell Proteomics (2021) 20:100113. doi: 10.1016/j.mcpro.2021.100113 34139362 PMC8205261

[119] Weber AG Chau AS Egeblad M Barnes BJ Janowitz T. Nebulized in-line endotracheal dornase Alfa and albuterol administered to mechanically ventilated COVID-19 patients: A case series. Mol Med (2020) 26:91. doi: 10.1186/s10020-020-00215-w 32993479 PMC7522910

[120] Toma A Darwish C Taylor M Harlacher J Darwish R. The use of dornase Alfa in the management of COVID-19-Associated adult respiratory distress syndrome. Crit Care Res Pract (2021) 2021:e8881115. doi: 10.1155/2021/8881115 PMC8074548 33986957

[121] Toy P Lowell C. TRALI - definition, mechanisms, incidence and clinical relevance. Best Pract Res Clin Anaesthesiol (2007) 21:183–93. doi: 10.1016/j.bpa.2007.01.003 PMC2767181 17650771

[122] Rebetz J Semple JW Kapur R. The pathogenic involvement of neutrophils in acute respiratory distress syndrome and transfusion-related acute lung injury. TMH (2018) 45:290–8. doi: 10.1159/000492950 PMC6257140 30498407

[123] Caudrillier A Kessenbrock K Gilliss BM Nguyen JX Marques MB Monestier M. Platelets induce neutrophil extracellular traps in transfusion-related acute lung injury. J Clin Invest (2012) 122:2661–71. doi: 10.1172/JCI61303 PMC3386815 22684106

[124] Thomas GM Carbo C Curtis BR Martinod K Mazo IB Schatzberg D. Extracellular DNA traps are associated with the pathogenesis of TRALI in humans and mice. Blood (2012) 119:6335–43. doi: 10.1182/blood-2012-01-405183 PMC3383196 22596262

[125] Adrover JM Carrau L Daßler-Plenker J Bram Y Chandar V Houghton S. Disulfiram inhibits neutrophil extracellular trap formation and protects rodents from acute lung injury and SARS-CoV-2 infection. JCI Insight (2022) 7. doi: 10.1172/jci.insight.157342 PMC8983145 35133984

[126] Looney MR Nguyen JX Hu Y Van Ziffle JA Lowell CA Matthay MA. Platelet depletion and aspirin treatment protect mice in a two-event model of transfusion-related acute lung injury. J Clin Invest (2009) 119:3450–61. doi: 10.1172/JCI38432 PMC2769181 19809160

[127] Hakkim A Fuchs TA Martinez NE Hess S Prinz H Zychlinsky A. Activation of the raf-MEK-ERK pathway is required for neutrophil extracellular trap formation. Nat Chem Biol (2011) 7:75–7. doi: 10.1038/nchembio.496 21170021

[128] Weyker PD Webb CAJ Kiamanesh D Flynn BC. Lung ischemia reperfusion injury: A bench-To-Bedside review. Semin Cardiothorac Vasc Anesth (2013) 17:28–43. doi: 10.1177/1089253212458329 23042205

[129] den Hengst WA Gielis JF Lin JY Van Schil PE De Windt LJ Moens AL. Lung ischemia-reperfusion injury: A molecular and clinical view on a complex pathophysiological process. Am J Physiol Heart Circ Physiol (2010) 299(5):H1283–99. doi: 10.1152/ajpheart.00251.2010 20833966

[130] Steimle CN Guynn TP Morganroth ML Bolling SF Carr K Deeb GM. Neutrophils are not necessary for ischemia-reperfusion lung injury. Ann Thorac Surg (1992) 53:64–72. doi: 10.1016/0003-4975(92)90758-v 1728243

[131] Deeb GM Grum CM Lynch MJ Guynn TP Gallagher KP Ljungman AG. Neutrophils are not necessary for induction of ischemia-reperfusion lung injury. J Appl Physiol (1985) (1990) 68:374–81. doi: 10.1152/jappl.1990.68.1.374 2312480

[132] de Perrot M Liu M Waddell TK Keshavjee S. Ischemia–reperfusion–induced lung injury. Am J Respir Crit Care Med (2003) 167:490–511. doi: 10.1164/rccm.200207-670SO 12588712

[133] Eppinger MJ Jones ML Deeb GM Bolling SF Ward PA. Pattern of injury and the role of neutrophils in reperfusion injury of rat lung. J Surg Res (1995) 58:713–8. doi: 10.1006/jsre.1995.1112 7791351

[134] Fiser SM Tribble CG Long SM Kaza AK Cope JT Laubach VE. Lung transplant reperfusion injury involves pulmonary macrophages and circulating leukocytes in a biphasic response. J Thorac Cardiovasc Surg (2001) 121:1069–75. doi: 10.1067/mtc.2001.113603 11385373

[135] Sayah DM Mallavia B Liu F Ortiz-Muñoz G Caudrillier A DerHovanessian A. Neutrophil extracellular traps are pathogenic in primary graft dysfunction after lung transplantation. Am J Respir Crit Care Med (2015) 191:455–63. doi: 10.1164/rccm.201406-1086OC PMC4351593 25485813

[136] Scozzi D Wang X Liao F Liu Z Zhu J Pugh K. Neutrophil extracellular trap fragments stimulate innate immune responses that prevent lung transplant tolerance. Am J Transplant (2019) 19:1011–23. doi: 10.1111/ajt.15163 PMC6438629 30378766

[137] Zhu S Yu Y Ren Y Xu L Wang H Ling X. The emerging roles of neutrophil extracellular traps in wound healing. Cell Death Dis (2021) 12:984. doi: 10.1038/s41419-021-04294-3 34686654 PMC8536667

[138] Schauer C Janko C Munoz LE Zhao Y Kienhöfer D Frey B. Aggregated neutrophil extracellular traps limit inflammation by degrading cytokines and chemokines. Nat Med (2014) 20:511–7. doi: 10.1038/nm.3547 24784231

[139] Hahn J Schauer C Czegley C Kling L Petru L Schmid B. Aggregated neutrophil extracellular traps resolve inflammation by proteolysis of cytokines and chemokines and protection from antiproteases. FASEB J (2019) 33:1401–14. doi: 10.1096/fj.201800752R PMC6355082 30130433

[140] Knopf J Leppkes M Schett G Herrmann M Muñoz LE. Aggregated NETs sequester and detoxify extracellular histones. Front Immunol (2019) 10:2176. doi: 10.3389/fimmu.2019.02176 31572386 PMC6749074

[141] Kaczmarek A Vandenabeele P Krysko DV. Necroptosis: The release of damage-associated molecular patterns and its physiological relevance. Immunity (2013) 38:209–23. doi: 10.1016/j.immuni.2013.02.003 23438821

[142] Scozzi D Ibrahim M Liao F Lin X Hsiao H-M Hachem R. Mitochondrial damage associated molecular patterns released by lung transplants are associated with primary graft dysfunction. Am J Transplant (2019) 19:1464–77. doi: 10.1111/ajt.15232 PMC6482093 30582269

[143] Carrasco Loza R Villamizar Rodríguez G Medel Fernández N. Ventilator-induced lung injury (VILI) in acute respiratory distress syndrome (ARDS): Volutrauma and molecular effects. Open Respir Med J (2015) 9:112–9. doi: 10.2174/1874306401509010112 PMC4541417 26312103

[144] Choudhury S Wilson MR Goddard ME O’Dea KP Takata M. Mechanisms of early pulmonary neutrophil sequestration in ventilator-induced lung injury in mice. Am J Physiology-Lung Cell Mol Physiol (2004) 287:L902–10. doi: 10.1152/ajplung.00187.2004 15257987

[145] Belperio JA Keane MP Burdick MD Londhe V Xue YY Li K. Critical role for CXCR2 and CXCR2 ligands during the pathogenesis of ventilator-induced lung injury. J Clin Invest (2002) 110:1703–16. doi: 10.1172/JCI15849 PMC151632 12464676

[146] Bohrer B Silveira RC Neto EC Procianoy RS. Mechanical ventilation of newborns infant changes in plasma pro- and anti-inflammatory cytokines. J Pediatr (2010) 156:16–9. doi: 10.1016/j.jpeds.2009.07.027 19783005

[147] Bose CL Laughon MM Allred EN O’Shea TM Van Marter LJ Ehrenkranz RA. Systemic inflammation associated with mechanical ventilation among extremely preterm infants. Cytokine (2013) 61:315–22. doi: 10.1016/j.cyto.2012.10.014 PMC3518391 23148992

[148] Keshari RS Jyoti A Dubey M Kothari N Kohli M Bogra J. Cytokines induced neutrophil extracellular traps formation: Implication for the inflammatory disease condition. PloS One (2012) 7:e48111. doi: 10.1371/journal.pone.0048111 23110185 PMC3482178

[149] Yildiz C Palaniyar N Otulakowski G Khan MA Post M Kuebler WM. Mechanical ventilation induces neutrophil extracellular trap formation. Anesthesiology (2015) 122:864–75. doi: 10.1097/ALN.0000000000000605 25665049

[150] Rossaint J Herter JM Van Aken H Napirei M Döring Y Weber C. Synchronized integrin engagement and chemokine activation is crucial in neutrophil extracellular trap–mediated sterile inflammation. Blood (2014) 123:2573–84. doi: 10.1182/blood-2013-07-516484 24335230

[151] Li H Pan P Su X Liu S Zhang L Wu D. Neutrophil extracellular traps are pathogenic in ventilator-induced lung injury and partially dependent on TLR4. BioMed Res Int (2017) 2017:8272504. doi: 10.1155/2017/8272504 29387725 PMC5745654

[152] Lord JM Midwinter MJ Chen Y-F Belli A Brohi K Kovacs EJ. The systemic immune response to trauma: An overview of pathophysiology and treatment. Lancet (2014) 384:1455–65. doi: 10.1016/S0140-6736(14)60687-5 PMC4729362 25390327

[153] Watkins TR Nathens AB Cooke CR Psaty BM Maier RV Cuschieri J. Acute respiratory distress syndrome after trauma: Development and validation of a predictive model. Crit Care Med (2012) 40:2295–303. doi: 10.1097/CCM.0b013e3182544f6a PMC3400931 22809905

[154] Pillay J Hietbrink F Koenderman L Leenen LPH. The systemic inflammatory response induced by trauma is reflected by multiple phenotypes of blood neutrophils. Injury (2007) 38:1365–72. doi: 10.1016/j.injury.2007.09.016 18061190

[155] Li X Luck ME Herrnreiter CJ Cannon AR Choudhry MA. IL-23 promotes neutrophil extracellular trap formation and bacterial clearance in a mouse model of alcohol and burn injury. ImmunoHorizons (2022) 6:64–75. doi: 10.4049/immunohorizons.2100109 35058308 PMC13560771

[156] Surolia R Li FJ Wang Z Kashyap M Srivastava RK Traylor AM. NETosis in the pathogenesis of acute lung injury following cutaneous chemical burns. JCI Insight (2021) 6(10):e147564. doi: 10.1172/jci.insight.147564 PMC8262367 34027893

[157] Laggner M Lingitz M-T Copic D Direder M Klas K Bormann D. Severity of thermal burn injury is associated with systemic neutrophil activation. Sci Rep (2022) 12:1654. doi: 10.1038/s41598-022-05768-w 35102298 PMC8803945

[158] Calfee CS Eisner MD Ware LB Thompson BT Parsons PE Wheeler AP. Trauma-associated lung injury differs clinically and biologically from acute lung injury due to other clinical disorders. Crit Care Med (2007) 35:2243–50. doi: 10.1097/01.CCM.0000280434.33451.87 PMC2765812 17944012

[159] Treggiari MM Hudson LD Martin DP Weiss NS Caldwell E Rubenfeld G. Effect of acute lung injury and acute respiratory distress syndrome on outcome in critically ill trauma patients. Crit Care Med (2004) 32:327–31. doi: 10.1097/01.CCM.0000108870.09693.42 14758144

[160] Bakowitz M Bruns B McCunn M. Acute lung injury and the acute respiratory distress syndrome in the injured patient. Scandinavian J Trauma Resuscitation Emergency Med (2012) 20:54. doi: 10.1186/1757-7241-20-54 PMC3518173 22883052

[161] Salim A Martin M Constantinou C Sangthong B Brown C Kasotakis G. Acute respiratory distress syndrome in the trauma intensive care unit: Morbid but not mortal. Arch Surg (2006) 141:655–8. doi: 10.1001/archsurg.141.7.655 16847235

[162] Fuchs A Monlish DA Ghosh S Chang S-W Bochicchio GV Schuettpelz LG. Trauma induces emergency hematopoiesis through IL-1/MyD88 dependent production of G-CSF. J Immunol (2019) 202:3020–32. doi: 10.4049/jimmunol.1801456 PMC6504560 30988118

[163] Hampson P Dinsdale RJ Wearn CM Bamford AL Bishop JRB Hazeldine J. Neutrophil dysfunction, immature granulocytes, and cell-free DNA are early biomarkers of sepsis in burn-injured patients: A prospective observational cohort study. Ann Surg (2017) 265:1241–9. doi: 10.1097/SLA.0000000000001807 27232244

[164] Hazeldine J Dinsdale RJ Naumann DN Acharjee A Bishop JRB Lord JM. Traumatic injury is associated with reduced deoxyribonuclease activity and dysregulation of the actin scavenging system. Burns Trauma (2021) 9:tkab001. doi: 10.1093/burnst/tkab001 33834079 PMC8014516

[165] Hesselink L Spijkerman R van Wessem KJP Koenderman L Leenen LPH Huber-Lang M. Neutrophil heterogeneity and its role in infectious complications after severe trauma. World J Emergency Surg (2019) 14:24. doi: 10.1186/s13017-019-0244-3 PMC6542247 31164913

[166] Janicova A Becker N Xu B Simic M Noack L Wagner N. Severe traumatic injury induces phenotypic and functional changes of neutrophils and monocytes. J Clin Med (2021) 10:4139. doi: 10.3390/jcm10184139 34575249 PMC8467869

[167] Mortaz E Zadian SS Shahir M Folkerts G Garssen J Mumby S. Does neutrophil phenotype predict the survival of trauma patients? Front Immunol (2019) 10:2122. doi: 10.3389/fimmu.2019.02122 31552051 PMC6743367

[168] Hazeldine J Dinsdale RJ Harrison P Lord JM. Traumatic injury and exposure to mitochondrial-derived damage associated molecular patterns suppresses neutrophil extracellular trap formation. Front Immunol (2019) 10:685. doi: 10.3389/fimmu.2019.00685 31001279 PMC6455291

[169] Itagaki K Kaczmarek E Kwon WY Chen L Vlková B Zhang Q. FPR1 blockade prevents receptor regulation by mitochondrial DAMPs and preserves neutrophil function after trauma. Crit Care Med (2020) 48:e123–32. doi: 10.1097/CCM.0000000000004094 PMC7337247 31939811

[170] Hazeldine J Hampson P Opoku FA Foster M Lord JM. N-formyl peptides drive mitochondrial damage associated molecular pattern induced neutrophil activation through ERK1/2 and P38 MAP kinase signalling pathways. Injury (2015) 46:975–84. doi: 10.1016/j.injury.2015.03.028 25817163

[171] Raoof M Zhang Q Itagaki K Hauser CJ. Mitochondrial peptides are potent immune activators that activate human neutrophils *Via* FPR-1. J Trauma Acute Care Surg (2010) 68:1328–34. doi: 10.1097/TA.0b013e3181dcd28d 20539176

[172] Itagaki K Riça I Konecna B Kim HI Park J Kaczmarek E. Role of mitochondria-derived danger signals released after injury in systemic inflammation and sepsis. Antioxid Redox Signal (2021) 35:1273–90. doi: 10.1089/ars.2021.0052 PMC8905257 33847158

[173] Dinsdale RJ Hazeldine J Al Tarrah K Hampson P Devi A Ermogenous C. Dysregulation of the actin scavenging system and inhibition of DNase activity following severe thermal injury. Br J Surg (2020) 107:391–401. doi: 10.1002/bjs.11310 31502663 PMC7079039

[174] Meng W Paunel-Görgülü A Flohé S Witte I Schädel-Höpfner M Windolf J. Deoxyribonuclease is a potential counter regulator of aberrant neutrophil extracellular traps formation after major trauma. Mediators Inflamm (2012) 2012:e149560. doi: 10.1155/2012/149560 PMC3270459 22315507

[175] Mutua V Gershwin LJ. A review of neutrophil extracellular traps (NETs) in disease: Potential anti-NETs therapeutics. Clinic Rev Allerg Immunol (2021) 61:194–211. doi: 10.1007/s12016-020-08804-7 PMC7395212 32740860

[176] Martinod K Wagner DD. Thrombosis: Tangled up in NETs. Blood (2014) 123:2768–76. doi: 10.1182/blood-2013-10-463646 PMC4007606 24366358

